# Supplementary material for: Protocol Development of a Personalized Balanced Nutrition Concept for Preschool Children, Primarily Those with Food Allergies, Using an IT Platform
Source: Medicina (Kaunas). 2023 Jul 26;59(8):1367. doi: 10.3390/medicina59081367 (PMC10456309; doi:10.3390/medicina59081367)
Supplement: Supplementary file 1 [file medicina-59-01367-s001.zip › medicina-2458568-supplementary.pdf]

Supplementary data for Miletić Gospić et al.

Table S1. List of all inhaled and food allergens used in standard SPT in participants

Type of allergen	Allergen species	Binomial nomenclature
House dust	House dust mite	*Dermatophagoides farinae*
		Dermatophagoides pteronyssinus
Animal dander	Cat dander	*Felis domesticus*
	Dog dander	*Canis familiaris*
	5 grasses mix- cocksfoot, sweet vernal-grass, rye-grass, meadow grass, and timothy	*Dactylis glomerata, Anthoxanthum odoratu*m, *Lolium perenne, Poa pratensis* and *Phleum pratense*
Weed pollen	Common ragweed	*Ambrosia elatior*
	Mugwort	*Artemisia vulgaris*
	Lichwort	*Parietaria officinalis*
Tree pollen	Common silver birch	*Betula verrucosa*
	Hazel	*Corylus avellana*
	Olive	*Olea europaea*
	Pine	*Pinus radiata*
	Cypress	*Cupressus sempervirens*
Shrub pollen	Mimosa	*Mimosa pudica*
Molds	Alternaria	*Alternaria alternata*
	Cladosporium	*Cladosporium herbarum* or *spp.*
Food allergens	Whole egg	*Gallus spp.*
	Cow`s milk	*Bos spp.*
	Wheat flour	*Triticum aestivum*
	Corn flour	*Zea mays*
	Soy	*Glycine max (Soja hispida)*
	Peanut	*Arachis hypogaea*
	Sesame	*Sesamum indicum*
	Hake	*Merluccius merluccius*
	Trout	*Salmo trutta fario*

	Hazelnut	*Corylus avellana*
	Walnut	*Juglans spp.*
	Almond	*Amygdalus communis*
	Cacao	*Theobroma cacao*
	Rice	*Oryza sativa*
